# Subject Index: Volume 11

**DOI:** 10.1111/jdi.13415

**Published:** 2020-11-04

1‐h plasma glucose levels

Characteristics associated with elevated 1‐h plasma glucose levels during a 75‐g oral glucose tolerance test in non‐obese Japanese men, Sato 1520–1523

25‐Hydroxyvitamin D

Association between serum 25‐hydroxyvitamin D concentrations and metabolic syndrome in the middle‐aged and elderly Chinese population in Dalian, northeast China: A cross‐sectional study, Weldegiorgis 184–191

α‐Gustducin

Characterization of the taste receptor‐related G‐protein, α‐gustducin, in pancreatic β‐cells, Udagawa 814–822

α‐Klotho

Protective potential of klotho protein on diabetic retinopathy: Evidence from clinical and *in vitro* studies, Ji 162–169

β‐Cell autoimmunity

Juvenile case with the coexistence of maturity‐onset diabetes of the young 1 and later‐onset latent autoimmune diabetes in youth, Yoshida 1010–1013

β‐Cell function

Longitudinal examination of pancreatic β‐cell function in Japanese individuals, Fujikawa 70–74
Proinsulin is sensitive to reflect glucose intolerance, Nakamura 75–79
Family history of diabetes in both parents is strongly associated with impaired residual β‐cell function in Japanese type 2 diabetes patients, Iwata 564–572
Pancreatic fat accumulation evaluated by multidetector computed tomography in patients with type 2 diabetes, Fukase 1188–1196

β‐Cell mass

Association of glucose tolerance status with pancreatic β‐ and α‐cell mass in community‐based autopsy samples of Japanese individuals: The Hisayama Study, Inaishi 1197–1206
Association of glucagon‐like peptide‐1 receptor‐targeted imaging probe with in vivo glucagon‐like peptide‐1 receptor agonist glucose‐lowering effects, Murakami 1448–1456

*ABCC8* gene

Identification of a compound heterozygous inactivating *ABCC8* gene mutation responsible for young‐onset diabetes with exome sequencing, Matsutani 333–336
Neonatal diabetes caused by the heterozygous Pro1198Leu mutation in the ABCC8 gene in a male infant: 6‐year clinical course, Uraki 502–505

Acarbose

Impact of baseline characteristics on glycemic effects of add‐on saxagliptin or acarbose to metformin therapy: Subgroup analysis of the SMART study in Chinese patients with type 2 diabetes mellitus, Fang 896–905

Acute coronary syndrome

Absolute risk of acute coronary syndrome after severe hypoglycemia: A population‐based 2‐year cohort study using the National Database in Japan, Nishioka 426–434

Adenosine monophosphate‐activated protein kinase

Impaired nicotinamide adenine dinucleotide (NAD+) metabolism in diabetes and diabetic tissues: Implications for nicotinamide‐related compound treatment, Fan 1403–1419

Adiponectin

Incidence of severe hypoglycemia and its association with serum adiponectin in Japanese patients with type 1 and insulin‐treated type 2 diabetes: The Fukuoka Diabetes Registry, Iwase 1258‐1264

Adipose‐derived mesenchymal stem cell

Transplantation of adipose‐derived mesenchymal stem cell sheets directly into the kidney suppresses the progression of renal injury in a diabetic nephropathy rat model, Takemura 545–553

Adrenomedullin

Adrenomedullin has a cytoprotective role against endoplasmic reticulum stress for pancreatic β‐cells in autocrine and paracrine manners, Suetomi 823–833

Aged society

New perspectives on insulin therapy, Araki 795–797

Air

Association between environmental quality and diabetes in the USA, Jagai 315–324

Alcohol consumption

Alcohol‐induced impaired insulin secretion in a Japanese population: 5‐year follow up in the Gifu Diabetes Study, Ueda 1207–1214

Amino acids

Consistency of plasma glucagon levels in patients with type 1 diabetes after a 1‐year period, Kawamori 337–340

Amyloid

Insulin‐derived amyloidosis without a palpable mass at the insulin injection site: A report of two cases, Nagase 1002–1005

Anaplastic lymphoma kinase

Ceritinib‐associated hyperglycemia in the Japanese Adverse Drug Event Report Database, Fujita 726–730

Anemia

Associations of anemia and hemoglobin with hemoglobin A1c among non‐diabetic workers in Japan, Sakamoto 719–725

Angiotensin‐converting enzyme

N‐acetyl‐seryl‐aspartyl‐lysyl‐proline is a valuable endogenous antifibrotic peptide for kidney fibrosis in diabetes: An update and translational aspects, Kanasaki 516–526

Antidiabetic drugs

Sodium–glucose cotransporters: Functional properties and pharmaceutical potential, Sano 770–782

Anti‐programmed death 1 therapy

Simultaneous development of Graves’ disease and type 1 diabetes during anti‐programmed cell death‐1 therapy: A case report, Kurihara 1006–1009

Aorta

Electronegative low‐density lipoprotein of patients with metabolic syndrome induces pathogenesis of aorta through disruption of the stimulated by retinoic acid 6 cascade, Chen 535–544

Apolipoprotein M

Apolipoprotein M overexpression through adeno‐associated virus gene transfer improves insulin secretion and insulin sensitivity in Goto‐Kakizaki rats, Yu 1150–1158

Apoptosis

Protective potential of klotho protein on diabetic retinopathy: Evidence from clinical and *in vitro* studies, Ji 162–169
Adrenomedullin has a cytoprotective role against endoplasmic reticulum stress for pancreatic β‐cells in autocrine and paracrine manners, Suetomi 823–833

Arterial stiffness

Association between mean platelet volume in the pathogenesis of type 2 diabetes mellitus and diabetic macrovascular complications in Japanese patients, Inoue 938–945

Asprosin

Increased serum level and impaired response to glucose fluctuation of asprosin is associated with type 2 diabetes mellitus, Zhang 349–355

Autoantibody

Characterization of patients with diabetes who were incidentally found to be glutamic acid decarboxylase autoantibody‐positive by bridging‐type enzyme‐linked immunosorbent assay, Kawasaki 1507–1510

Autoimmune gastritis

Serum chromogranin A level continuously rises with the progression of type 1 diabetes, and indicates the presence of both enterochromaffin‐like cell hyperplasia and autoimmune gastritis, Herold 865‐873
Statin‐induced autoimmune hepatitis in patients with type 1 diabetes: A report of two cases and literature review, Kawasaki 1673–1676

Baba’s classification

*Lumos* for the long trail: Strategies for clinical diagnosis and severity staging for diabetic polyneuropathy and future directions, Himeno 5–16

Berberine

Berberine ameliorates renal impairment and inhibits podocyte dysfunction by targeting the phosphatidylinositol 3‐kinase–protein kinase B pathway in diabetic rats, Ni 297–306

Bicarbonate ions

Low urine pH predicts new onset of diabetes mellitus during a 10‐year period in men: BOREAS‐DM1 study, Higashiura 1490–1497

Bioavailable testosterone

What is the best biological parameter to predict erectile dysfunction in men aged >55 years with type 2 diabetes?, Raharinavalona 170–173

Biomarker

Circulating kidney injury molecule‐1 as a biomarker of renal parameters in diabetic kidney disease, Gohda 435–440
Protective factors as biomarkers and targets for prevention and treatment of diabetic nephropathy: From current human evidence to future possibilities, Nowak 1085–1096

Birthweight

Low birthweight is associated with type 2 diabetes mellitus in Japanese adults: The Toon Health Study, Yokoyama 1643–1650

Body mass index

Distinct developmental trajectories of body mass index and diabetes risk: A 5‐year longitudinal study of Chinese adults, Dai 466–474
Impact of physical activity and sedentary time on glycated hemoglobin levels and body composition: Cross‐sectional study using outpatient clinical data of Japanese patients with type 2 diabetes, Nakanishi 633–639
Is visceral abdominal fat area a better indicator for hyperglycemic risk? Results from the Pinggu Metabolic Disease Study, Zhang 888–895

Bodyweight change

Bodyweight threshold for sudden onset of ketosis might exist in ketosis‐prone type 2 diabetes patients, Satomura 499–501

Bone mineral density

Association between the Geriatric Nutritional Risk Index, bone mineral density and osteoporosis in type 2 diabetes patients, Wang 956–963

Breast cancer

Increasing risk of diabetes mellitus in postmenopausal women with newly diagnosed primary breast cancer, Wang 490–498

Cancer

Cancer has overtaken cardiovascular disease as the commonest cause of death in Scottish type 2 diabetes patients: A population‐based study (The Ayrshire Diabetes Follow‐up Cohort study), Collier 55–61
Prevalence of diabetes in Japanese patients with cancer, Saito 1159–1162
Healthcare utilization and direct medical cost in the years during and after cancer diagnosis in patients with type 2 diabetes mellitus, Wu 1661–1672

Cancer antigen 19‐9

Association between cancer antigen 19‐9 and diabetes risk: A prospective and Mendelian randomization study, Li 585–593

Capillary electrophoresis

Silent hemoglobin variant during capillary electrophoresis: A case report, Yuan 1014–1017

Cardiac remodeling

Protective effects of glucagon‐like peptide‐1 on cardiac remodeling by inhibiting oxidative stress through mammalian target of rapamycin complex 1/p70 ribosomal protein S6 kinase pathway in diabetes mellitus, Wang 39–51

Cardiovascular autonomic neuropathy

Hypoglycemia unawareness and autonomic dysfunction in diabetes: Lessons learned and roles of diabetes technologies, Lin 1388–1402

Cardiovascular disease

Secondary analyses to assess the profound effects of empagliflozin on endothelial function in patients with type 2 diabetes and established cardiovascular diseases: The placebo‐controlled double‐blind randomized effect of empagliflozin on endothelial function in cardiovascular high risk diabetes mellitus: Multi‐center placebo‐controlled double‐blind randomized trial, Tanaka 1551–1563

Cardiovascular outcome

Cardiovascular outcomes of vildagliptin in patients with type 2 diabetes mellitus after acute coronary syndrome or acute ischemic stroke, Chen 110–124

Cardiovascular risks

Higher triglyceride to high‐density lipoprotein cholesterol ratio increases cardiovascular risk: 10‐year prospective study in a cohort of Chinese adults, Chen 475–481

Causes of deaths

Causes of death and estimated life expectancy among people with diabetes: A retrospective cohort study in a diabetes clinic, Goto 52–54

Cell cycle

Activation of overexpressed glucagon‐like peptide‐1 receptor attenuates prostate cancer growth by inhibiting cell cycle progression, Shigeoka 1137–1149

Cell sheet

Transplantation of adipose‐derived mesenchymal stem cell sheets directly into the kidney suppresses the progression of renal injury in a diabetic nephropathy rat model, Takemura 545–553

Ceramide

Analysis of urinary sphingolipids using liquid chromatography‐tandem mass spectrometry in diabetic nephropathy, Morita 441–449

Ceritinib

Ceritinib‐associated hyperglycemia in the Japanese Adverse Drug Event Report Database, Fujita 726–730

Child

Corneal nerve loss in children with type 1 diabetes mellitus without retinopathy or microalbuminuria, Gad 1594–1601

Childbirth

Association between age at first childbirth and type 2 diabetes in Chinese women, Qu 223–231

China

Association between age at first childbirth and type 2 diabetes in Chinese women, Qu 223–231
Excessive gestational weight gain in the first and second trimester is a risk factor for gestational diabetes mellitus among women pregnant with singletons: A repeated measures analysis, Qi 1651–1660

Chinese

Efficacy and safety of dulaglutide monotherapy compared with glimepiride in Chinese patients with type 2 diabetes: Post‐hoc analyses of a randomized, double‐blind, phase III study, Shi 142–150
Higher triglyceride to high‐density lipoprotein cholesterol ratio increases cardiovascular risk: 10‐year prospective study in a cohort of Chinese adults, Chen 475–481
Factors associated with glycemic control in type 1 diabetes patients in China: A cross‐sectional study, Huo 1575–1582

Chinese male

Triglyceride to high‐density lipoprotein cholesterol ratio is associated with incident diabetes in men: A retrospective study of Chinese individuals, Qin 192–198

Chinese patients

Identification of insulin gene variants in patients with neonatal diabetes in the Chinese population, Fu 578–584

Chromogranin A

Serum chromogranin A level continuously rises with the progression of type 1 diabetes, and indicates the presence of both enterochromaffin‐like cell hyperplasia and autoimmune gastritis, Herold 865–873

Chronic kidney failure

Efficacy and safety of trelagliptin in Japanese patients with type 2 diabetes with severe renal impairment or end‐stage renal disease: Results from a randomized, phase 3 study, Kaku 373–381

Clinical effectiveness

Evaluation of effectiveness of treatment paradigm for newly diagnosed type 2 diabetes patients in China: A nationwide prospective cohort study, Cai 151–161

Cohort

Time course of metabolic status in pregnant women: The Japan Environment and Children’s Study, Sasaki 1318–1325

Commentary

Evidence‐based comparison of glucagon‐like peptide receptor agonists and sodium–glucose cotransporter 2 inhibitors, Watada 17–19
Detailed analysis at a single‐cell level of cells undergoing pancreatic differentiation, Shiraki 20–21
Combined effect of sodium–glucose cotransporter 2 and dipeptidyl peptidase‐4 inhibitors for diabetic kidney disease, Araki 22–24
Pancreatic β‐cells express major histocompatibility complex class II: Do diabetic β‐cells have the capacity of antigen‐presenting cells?, Sano 281–283
β‐Cell senescence in the pathogenesis of type 2 diabetes, Imai 284–286
Commentary on the effects of receptor activator of nuclear factor‐B ligand inhibition on bone mass and muscle strength, Hwang 287–289
Fatty acid overload to compromised oxidative phosphorylation activates inflammation in type 2 diabetes: Hidden beasts and how to find them, Lee 290–293
CREDENCE: A silver lining in the dark cloud of diabetic nephropathy, Hanai 527–529
Incidence of severe urinary tract infections not increased by initiating sodium–glucose cotransporter 2 inhibitors, Yokoyama 530–531
Novel mechanism of increasing cerebral vascular constriction in acute hyperglycemia and diabetes through adenylyl cyclase 5‐generated cyclic adenosine monophosphate, Sakamoto 783–785
Are the polyol pathway and hyperuricemia partners in the development of non‐alcoholic fatty liver disease in diabetes?, Hotta 786–788
Potential linkage between dipeptidyl peptidase‐4 inhibitor use and the risk of pancreatitis/pancreatic cancer, Shirakawa 789–791
Does glycemic control rescue type 2 diabetes patients from COVID‐19‐related deaths?, Naruse 792–794
Lowering the risk of gout: Another benefits from the use of sodium–glucose cotransporter 2 inhibitors, Sheu 1115–1116
What can we learn from the islet of Joslin Medalists?, Yagihashi 1117–1119
Angiotensin‐converting enzyme 2 as a versatile player in the management of coronavirus disease 2019, Amano 1120–1122
Effects of metformin on blood glucose levels and bodyweight mediated through intestinal effects, Harada 1420–1421
Cardioprotective effects of GLP‐1(28‐36a): A degraded metabolite or GLP‐1’s better half?, Liu 1422–1425
Does the breakdown of the detoxification system for aldehydes as a result of aldose reductase upregulation lead to alcohol‐induced liver injury in humans and mice?, Hotta 1426–1430
Complement‐1q tumor necrosis factor‐α‐related protein isoform 5
Circulating complement‐1q tumor necrosis factor‐α‐related protein isoform 5 levels are low in type 2 diabetes patients and reduced by dapagliflozin, Zhang 88–95

Composite end‐point

Achieving the composite end‐point of glycated hemoglobin <7.0% without weight gain or hypoglycemia with once‐weekly dulaglutide in Chinese patients with type 2 diabetes: A post‐hoc analysis, Xiao 647–652

Conditioned medium

Secreted factors from cultured dental pulp stem cells promoted neurite outgrowth of dorsal root ganglion neurons and ameliorated neural functions in streptozotocin‐induced diabetic mice, Miura‐Yura 28–38

Continuous 13C breath test

Magnitude of slowing gastric emptying by glucagon‐like peptide‐1 receptor agonists determines the amelioration of postprandial glucose excursion in Japanese patients with type 2 diabetes, Suganuma 389–399

Continuous glucose monitoring

Relationship between glycated hemoglobin level and duration of hypoglycemia in type 2 diabetes patients treated with sulfonylureas: A multicenter cross‐sectional study, Matsuoka 417–425
Critical discrepancy in blood glucose control levels evaluated by glycated albumin and estimated hemoglobin A1c levels determined from a flash continuous glucose monitoring system in patients with type 2 diabetes on hemodialysis, Ushigome 1570–1574

Coping profile

Coping styles associated with glucose control in individuals with type 2 diabetes mellitus, Murakami 1215–1221

Cortical thickness

Inverse association of plasma leptin with cortical thickness at distal radius determined with a quantitative ultrasound device in patients with type 2 diabetes mellitus, Kurajoh 174–183

COVID‐19

Dissecting the interaction between COVID‐19 and diabetes mellitus, Chee 1104–1114
A territory‐wide study on the impact of COVID‐19 on diabetes‐related acute care, Lui 1303–1306

C‐reactive protein

Intra‐individual association between C‐reactive protein and insulin administration in postoperative lumbar spinal canal stenosis patients: A retrospective cohort study, Kurisu 980–984

Cross‐sectional study

Ratios of serum eicosapentaenoic acid to arachidonic acid and docosahexaenoic acid to arachidonic acid were inversely associated with serum resistin levels: The Hisayama Study, Higashioka 482–489
Standard medical nutrition therapy of 25 kcal/kg ideal bodyweight/day often does not reach even resting energy expenditure for patients with type 2 diabetes, Fukuda 626–632

Cumulative environmental exposures

Association between environmental quality and diabetes in the USA, Jagai 315–324

Death

Impact of hip fracture on all‐cause mortality in Japanese patients with type 2 diabetes mellitus: The Fukuoka Diabetes Registry, Komorita 62–69

Dehydration

Recommendations on the Proper Use of SGLT2 Inhibitors, The Committee on the Proper Use of SGLT2 Inhibitors 257–261

Dementia

Dementia risk by combinations of metabolic diseases and body mass index: Japan Gerontological Evaluation Study Cohort Study, Yokomichi 206–215
Elevated serum glycated albumin and glycated albumin : hemoglobin A1c ratio were associated with hippocampal atrophy in a general elderly population of Japanese: The Hisayama Study, Ohara 971–979

Diabetes

*Helicobacter pylori* infection is associated with diabetes among Chinese adults, Wan 199–205
Identification of a compound heterozygous inactivating *ABCC8* gene mutation responsible for young‐onset diabetes with exome sequencing, Matsutani 333–336
Three‐component non‐invasive risk score for undiagnosed diabetes in Chinese people: Development, validation and longitudinal evaluation, Woo 341–348
Absolute risk of acute coronary syndrome after severe hypoglycemia: A population‐based 2‐year cohort study using the National Database in Japan, Nishioka 426–434
Distinct developmental trajectories of body mass index and diabetes risk: A 5‐year longitudinal study of Chinese adults, Dai 466–474
Is visceral abdominal fat area a better indicator for hyperglycemic risk? Results from the Pinggu Metabolic Disease Study, Zhang 888–895
Japanese Clinical Practice Guideline for Diabetes 2019, Arak 1020–1076
Dissecting the interaction between COVID‐19 and diabetes mellitus, Chee 1104–1114
Apolipoprotein M overexpression through adeno‐associated virus gene transfer improves insulin secretion and insulin sensitivity in Goto‐Kakizaki rats, Yu 1150–1158
Prevalence of diabetes in Japanese patients with cancer, Saito 1159–1162
A territory‐wide study on the impact of COVID‐19 on diabetes‐related acute care, Lui 1303–1306
Time course of metabolic status in pregnant women: The Japan Environment and Children’s Study, Sasaki 1318–1325
A 50‐year history of the health impacts of Westernization on the lifestyle of Japanese Americans: A focus on the Hawaii–Los Angeles–Hiroshima Study, Yoneda 1382–1387

Diabetes complications

Transient receptor potential vanilloid 4 channels as therapeutic targets in diabetes and diabetes‐related complications, Hu 757–769

Diabetes mellitus

Protective effects of glucagon‐like peptide‐1 on cardiac remodeling by inhibiting oxidative stress through mammalian target of rapamycin complex 1/p70 ribosomal protein S6 kinase pathway in diabetes mellitus, Wang 39–51
Trends in the prevalence, awareness, treatment and control of diabetes in rural areas of northern China from 1992 to 2011, Zhang 241–249
Acute esophageal necrosis after cellulitis in an obese patient with diabetes mellitus, Tanaka 250–252
Increasing risk of diabetes mellitus in postmenopausal women with newly diagnosed primary breast cancer, Wang 490–498
Association between cancer antigen 19‐9 and diabetes risk: A prospective and Mendelian randomization study, Li 585–593
Opportunistic invasive fungal disease in patients with type 2 diabetes mellitus from Southern China: Clinical features and associated factors, Lao 731–744
Transient receptor potential vanilloid 4 channels as therapeutic targets in diabetes and diabetes‐related complications, Hu 757–769
Association between type 2 diabetes mellitus, especially recently uncontrolled glycemia, and intracranial plaque characteristics: A high‐resolution magnetic resonance imaging study, Huang 1278–1284
Passive smoking and type 2 diabetes among never‐smoking women: The Japan Public Health Center‐based Prospective Study, Oba 1352–1358
General glycosylated hemoglobin goals potentially increase myocardial infarction severity in diabetes patients with comorbidities: Insights from a nationwide multicenter study, Che 1498–1506

Diabetes risk score

Dysglycemia risk score in Saudi Arabia: A tool to identify people at high future risk of developing type 2 diabetes, Bahijri 844–855

Diabetic kidney disease

Prevalence of albuminuria and renal dysfunction, and related clinical factors in Japanese patients with diabetes: The Japan Diabetes Complication and its Prevention prospective study 5, Shikata 325–332
Circulating kidney injury molecule‐1 as a biomarker of renal parameters in diabetic kidney disease, Gohda 435–440
Decreased microcirculatory function measured by perfusion index is a novel indicator of diabetic kidney disease in patients with type 2 diabetes, Okada 681–687
Protective factors as biomarkers and targets for prevention and treatment of diabetic nephropathy: From current human evidence to future possibilities, Nowak 1085–1096
Combined effect of diabetic retinopathy and diabetic kidney disease on all‐cause, cancer, vascular and non‐cancer non‐vascular mortality in patients with type 2 diabetes: A real‐world longitudinal study, Takao 1170–1180
CD‐1*^db/db^* mice: A novel type 2 diabetic mouse model with progressive kidney fibrosis, Mizunuma 1470–1481

Diabetic myocardium

Activation of autophagy inhibits nucleotide‐binding oligomerization domain‐like receptor protein 3 inflammasome activation and attenuates myocardial ischemia‐reperfusion injury in diabetic rats, Zhang 1126–1136

Diabetic nephropathy

Prevalence of albuminuria and renal dysfunction, and related clinical factors in Japanese patients with diabetes: The Japan Diabetes Complication and its Prevention prospective study 5, Shikata 325–332
Opportunistic invasive fungal disease in patients with type 2 diabetes mellitus from Southern China: Clinical features and associated factors, Lao 731–744
Effects of ipragliflozin on the development and progression of kidney disease in patients with type 2 diabetes: An analysis from a multicenter prospective intervention study, Matsuba 1248–1257

Diabetic nephropathies

Association between Pro12Ala polymorphism and albuminuria in type 2 diabetic nephropathy, Chen 923–929

Diabetic peripheral neuropathic pain

Long‐term safety and efficacy of mirogabalin in Asian patients with diabetic peripheral neuropathic pain, Baba 693–698

Diabetic retinopathy

Protective potential of klotho protein on diabetic retinopathy: Evidence from clinical and *in vitro* studies, Ji 162–169
Vitreous hemorrhage in diabetes patients with proliferative diabetic retinopathy undergoing hemodialysis, Kameda 688–692
Effect of postprandial hyperglycemia at clinic visits on the incidence of retinopathy in patients with type 2 diabetes: An analysis using real‐world long‐term follow‐up data, Takao 930–937
Combined effect of diabetic retinopathy and diabetic kidney disease on all‐cause, cancer, vascular and non‐cancer non‐vascular mortality in patients with type 2 diabetes: A real‐world longitudinal study, Takao 1170–1180

Diabetic polyneuropathies

Early sensory neurophysiological changes in prediabetes, Lin 458–465

Diabetic polyneuropathy

*Lumos* for the long trail: Strategies for clinical diagnosis and severity staging for diabetic polyneuropathy and future directions, Himeno 5–16
Secreted factors from cultured dental pulp stem cells promoted neurite outgrowth of dorsal root ganglion neurons and ameliorated neural functions in streptozotocin‐induced diabetic mice, Miura‐Yura 28–38

Diabetic polyneuropathy‐related symptoms

Factors associated with diabetic polyneuropathy‐related sensory symptoms and signs in patients with polyneuropathy: A cross‐sectional Japanese study (JDDM 52) using a non‐linear model, Yokoyama 450–457

Diagnosis

Japanese Clinical Practice Guideline for Diabetes 2019, Arak 1020–1076
Diagnosing peripheral neuropathy in South‐East Asia: A focus on diabetic neuropathy, Malik 1097–1103

Dialysis

Vitreous hemorrhage in diabetes patients with proliferative diabetic retinopathy undergoing hemodialysis, Kameda 688–692

Dietary habit

Intake of sucrose affects gut dysbiosis in patients with type 2 diabetes, Hashimoto, 1623–1634

Direct medical costs

Healthcare utilization and direct medical cost in the years during and after cancer diagnosis in patients with type 2 diabetes mellitus, Wu 1661–1672

Dipeptidyl peptidase‐4 inhibitors

Factors associated with the glucose‐lowering efficacy of sitagliptin in Japanese patients with type 2 diabetes mellitus: Pooled analysis of Japanese clinical trials, Tajima 640–646

DPNCheck

*Lumos* for the long trail: Strategies for clinical diagnosis and severity staging for diabetic polyneuropathy and future directions, Himeno 5–16

Drug‐induced hyperglycemia

Ceritinib‐associated hyperglycemia in the Japanese Adverse Drug Event Report Database, Fujita 726–730

Dulaglutide

Efficacy and safety of dulaglutide monotherapy compared with glimepiride in Chinese patients with type 2 diabetes: Post‐hoc analyses of a randomized, double‐blind, phase III study, Shi 142–150
Achieving the composite end‐point of glycated hemoglobin <7.0% without weight gain or hypoglycemia with once‐weekly dulaglutide in Chinese patients with type 2 diabetes: A post‐hoc analysis, Xiao 647–652

Dysglycemia

Dysglycemia risk score in Saudi Arabia: A tool to identify people at high future risk of developing type 2 diabetes, Bahijri 844–855

Editorial

A new era for research publication: Will Open Access become the norm?, Hotta 3–4
New concept of the glucagon‐like peptide‐1 signaling pathway on pancreatic insulin secretion, Kaku 265–267
How do you make new β‐cells in humans? An attempt to create human β‐cells from α‐cells of brain‐dead donors, Fujitani 513–515
Dawn of a new era of diabeto‐oncology, Noto 755–756
The Asian Association for the Study of Diabetes: The first 10 years and the next 10 years, Seino 1079–1084
Low‐density lipoprotein cholesterol and stroke: How low should we go?, Lui 1379–1381

Elderly

Safety and effectiveness of tofogliflozin in elderly Japanese patients with type 2 diabetes mellitus: A subanalysis of a post‐marketing study (J‐STEP/EL Study), Kaku 405–416
Efficacy and safety of metformin and sitagliptin‐based dual and triple therapy in elderly Chinese patients with type 2 diabetes: Subgroup analysis of STRATEGY study, Liu 1532–1541

Electronegative low‐density lipoprotein

Electronegative low‐density lipoprotein of patients with metabolic syndrome induces pathogenesis of aorta through disruption of the stimulated by retinoic acid 6 cascade, Chen 535–544

Empagliflozin

Effects of liraglutide and empagliflozin added to insulin therapy in patients with type 2 diabetes: A randomized controlled study, Nakaguchi 1542–1550
Secondary analyses to assess the profound effects of empagliflozin on endothelial function in patients with type 2 diabetes and established cardiovascular diseases: The placebo‐controlled double‐blind randomized effect of empagliflozin on endothelial function in cardiovascular high risk diabetes mellitus: Multi‐center placebo‐controlled double‐blind randomized trial, Tanaka 1551–1563

Endoscopy

Acute esophageal necrosis after cellulitis in an obese patient with diabetes mellitus, Tanaka 250–252

Endothelial function

Secondary analyses to assess the profound effects of empagliflozin on endothelial function in patients with type 2 diabetes and established cardiovascular diseases: The placebo‐controlled double‐blind randomized effect of empagliflozin on endothelial function in cardiovascular high risk diabetes mellitus: Multi‐center placebo‐controlled double‐blind randomized trial, Tanaka 1551–1563

Endothelial–mesenchymal transition

*N*‐acetyl‐seryl‐aspartyl‐lysyl‐proline is a valuable endogenous antifibrotic peptide for kidney fibrosis in diabetes: An update and translational aspects, Kanasaki 516–526

Enlarged islets

Tumor‐like features of gene expression and metabolic profiles in enlarged pancreatic islets are associated with impaired incretin‐induced insulin secretion in obese diabetes: A study of Zucker fatty diabetes mellitus rat, Hayami 1434–1447

Enterochromaffin‐like cell hyperplasia

Serum chromogranin A level continuously rises with the progression of type 1 diabetes, and indicates the presence of both enterochromaffin‐like cell hyperplasia and autoimmune gastritis, Herold 865–873

Enzyme‐linked immunosorbent assay

Increased diagnosis of autoimmune childhood‐onset Japanese type 1 diabetes using a new glutamic acid decarboxylase antibody enzyme‐linked immunosorbent assay kit, compared with a previously used glutamic acid decarboxylase antibody radioimmunoassay kit, Sugihara 594–602

Epidemiology

Proinsulin is sensitive to reflect glucose intolerance, Nakamura 75–79
Trends in the prevalence, awareness, treatment and control of diabetes in rural areas of northern China from 1992 to 2011, Zhang 241–249

Erectile dysfunction

What is the best biological parameter to predict erectile dysfunction in men aged >55 years with type 2 diabetes?, Raharinavalona 170–173

Erythropoietin

Failure to confirm a sodium–glucose cotransporter 2 inhibitor‐induced hematopoietic effect in non‐diabetic rats with renal anemia, Yamazaki 834–843

Esophagitis

Acute esophageal necrosis after cellulitis in an obese patient with diabetes mellitus, Tanaka 250–252
Clinical and genetic analysis in a family with familial renal glucosuria: Identification of an N101K mutation in the sodium–glucose cotransporter 2 encoded by a solute carrier family 5 member 2 gene, Sada 573–577

Estimated hemoglobin A1c

Critical discrepancy in blood glucose control levels evaluated by glycated albumin and estimated hemoglobin A1c levels determined from a flash continuous glucose monitoring system in patients with type 2 diabetes on hemodialysis, Ushigome 1570–1574

Euglycemic ketoacidosis

Case report of superior mesenteric artery syndrome that developed in a lean type 2 diabetes patient and was associated with rapid body weight loss after sodium–glucose cotransporter 2 inhibitor administration, Hirai 1359–1362

Exercise

Effect of the use of passive body trunk exercise equipment on oxygen consumption and self‐efficacy for carrying out exercise in patients with type 2 diabetes, Kawae 1265–1271

False positive

Characterization of patients with diabetes who were incidentally found to be glutamic acid decarboxylase autoantibody‐positive by bridging‐type enzyme‐linked immunosorbent assay, Kawasaki 1507–1510

Familial renal glucosuria

Family history of diabetes in both parents is strongly associated with impaired residual β‐cell function in Japanese type 2 diabetes patients, Iwata 564–572

Family history of diabetes

Family history of diabetes in both parents is strongly associated with impaired residual β‐cell function in Japanese type 2 diabetes patients, Iwata 564–572

Fatty liver

Correlation between serum proinsulin levels and fatty liver: The Dynamics of Lifestyle and Neighborhood Community on Health Study Health Study, Miya 964–970

Ferritin

Association between the ferritin level and risk of gestational diabetes mellitus: A meta‐analysis of observational studies, Sun 707–718
Iron metabolism and type 2 diabetes mellitus: A meta‐analysis and systematic review, Liu 946–955

Fibroblast growth factor

*N*‐acetyl‐seryl‐aspartyl‐lysyl‐proline is a valuable endogenous antifibrotic peptide for kidney fibrosis in diabetes: An update and translational aspects, Kanasaki 516‐526

Fibrosis

CD‐1*^db/db^* mice: A novel type 2 diabetic mouse model with progressive kidney fibrosis, Mizunuma 1470–1481

Flash glucose monitoring

Flash glucose monitoring in type 1 diabetes: A comparison with self‐monitoring blood glucose, Babaya 1222–1229

Galectin‐3

Prevalence of mild cognitive impairment in type 2 diabetes mellitus is associated with serum galectin‐3 level, Ma 1295–1302

Gastric emptying

Magnitude of slowing gastric emptying by glucagon‐like peptide‐1 receptor agonists determines the amelioration of postprandial glucose excursion in Japanese patients with type 2 diabetes, Suganuma 389–399

Geriatric nutritional risk index

Association between the Geriatric Nutritional Risk Index, bone mineral density and osteoporosis in type 2 diabetes patients, Wang 956–963

Gestational diabetes

Impaired early‐phase suppression of glucagon secretion after glucose load is associated with insulin requirement during pregnancy in gestational diabetes, Horie 232–240

Gestational diabetes mellitus

Comparison of pregnancy outcomes between women with early‐onset and late‐onset gestational diabetes in a retrospective multi‐institutional study in Japan, Usami 216–222
Association between the ferritin level and risk of gestational diabetes mellitus: A meta‐analysis of observational studies, Sun 707–718
Long‐term maternal cardiometabolic outcomes 22 years after gestational diabetes mellitus, Tutino 985–993
Have pregnancy outcomes improved with the introduction of the International Association of Diabetes and Pregnancy Study Groups criteria in Japan?, Nakanishi 994–1001
Whole transcriptome expression profiles in placenta samples from women with gestational diabetes mellitus, Tang 1307–1317
High serum triglyceride levels in the early first trimester of pregnancy are associated with gestational diabetes mellitus: A prospective cohort study, Zhu 1635–1642
Excessive gestational weight gain in the first and second trimester is a risk factor for gestational diabetes mellitus among women pregnant with singletons: A repeated measures analysis, Qi 1651–1660

Gestational weight gain

Excessive gestational weight gain in the first and second trimester is a risk factor for gestational diabetes mellitus among women pregnant with singletons: A repeated measures analysis, Qi 1651–1660

Glucagon

Impaired early‐phase suppression of glucagon secretion after glucose load is associated with insulin requirement during pregnancy in gestational diabetes, Horie 232–240
Consistency of plasma glucagon levels in patients with type 1 diabetes after a 1‐year period, Kawamori 337–340

Glucagon‐like peptide‐1

Protective effects of glucagon‐like peptide‐1 on cardiac remodeling by inhibiting oxidative stress through mammalian target of rapamycin complex 1/p70 ribosomal protein S6 kinase pathway in diabetes mellitus, Wang 39–51

Glucagon‐like peptide‐1 receptor

Activation of overexpressed glucagon‐like peptide‐1 receptor attenuates prostate cancer growth by inhibiting cell cycle progression, Shigeoka 1137–1149
Association of glucagon‐like peptide‐1 receptor‐targeted imaging probe with *in vivo* glucagon‐like peptide‐1 receptor agonist glucose‐lowering effects, Murakami 1448–1456

Glucagon‐like peptide‐1 receptor agonist

Dulaglutide‐combined basal plus correction insulin therapy contributes to ideal glycemic control in non‐critical hospitalized patients, Fushimi 125–131
Efficacy and safety of once‐weekly exenatide after switching from twice‐daily exenatide in patients with type 2 diabetes, Watanabe 382–388
Association of glucagon‐like peptide‐1 receptor‐targeted imaging probe with *in vivo* glucagon‐like peptide‐1 receptor agonist glucose‐lowering effects, Murakami 1448–1456

Glucagon‐like peptide‐1 receptor agonists

Magnitude of slowing gastric emptying by glucagon‐like peptide‐1 receptor agonists determines the amelioration of postprandial glucose excursion in Japanese patients with type 2 diabetes, Suganuma 389–399

Glucose fluctuation

Increased serum level and impaired response to glucose fluctuation of asprosin is associated with type 2 diabetes mellitus, Zhang 349–355

Glucose intolerance

Different pathogenesis of glucose intolerance in two subtypes of primary aldosteronism: Aldosterone‐producing adenoma and idiopathic hyperaldosteronism, Okazaki‐Hada 1511–1519

Glutamic acid decarboxylase

Clinical and genetic characteristics of people with type 1 diabetes who have discrepancies in titers of anti‐glutamic acid decarboxylase antibody measured by radioimmunoassay and enzyme‐linked immunosorbent assay, Takagi 356–362

Glutamic decarboxylase antibody

Increased diagnosis of autoimmune childhood‐onset Japanese type 1 diabetes using a new glutamic acid decarboxylase antibody enzyme‐linked immunosorbent assay kit, compared with a previously used glutamic acid decarboxylase antibody radioimmunoassay kit, Sugihara 594–602

Glycated hemoglobin

Impact of physical activity and sedentary time on glycated hemoglobin levels and body composition: Cross‐sectional study using outpatient clinical data of Japanese patients with type 2 diabetes, Nakanishi 633–639
Silent hemoglobin variant during capillary electrophoresis: A case report, Yuan 1014–1017
Increased risks of different grades of non‐alcoholic fatty liver disease in prediabetic subjects with impaired fasting glucose and glucose tolerance, including the isolated glycosylated hemoglobin levels of 5.7–6.4% in a Chinese population, Li 1336–1343

Glycemic control

Coping styles associated with glucose control in individuals with type 2 diabetes mellitus, Murakami 1215–1221
Factors associated with glycemic control in type 1 diabetes patients in China: A cross‐sectional study, Huo 1575–1582

Glycemic metabolism

*Helicobacter pylori* infection is associated with diabetes among Chinese adults, Wan 199–205

Glycosuria

Elevation of the renal threshold for glucose is associated with insulin resistance and higher glycated hemoglobin levels, Hieshima 617–625

Glycosylated hemoglobin

General glycosylated hemoglobin goals potentially increase myocardial infarction severity in diabetes patients with comorbidities: Insights from a nationwide multicenter study, Che 1498–1506

Graves’ disease

Simultaneous development of Graves’ disease and type 1 diabetes during anti‐programmed cell death‐1 therapy: A case report, Kurihara 1006–1009

Gut microbiota

Association between gut microbiota composition and glycoalbumin level during pregnancy in Japanese women: Pilot study from Chiba Study of Mother and Child Health, Sakurai 699–706
Intake of sucrose affects gut dysbiosis in patients with type 2 diabetes, Hashimoto, 1623–1634

Guideline

Japanese Clinical Practice Guideline for Diabetes 2019, Arak 1020–1076

*Helicobacter pylori* infection

*Helicobacter pylori* infection is associated with diabetes among Chinese adults, Wan 199–205

Hemodialysis

Critical discrepancy in blood glucose control levels evaluated by glycated albumin and estimated hemoglobin A1c levels determined from a flash continuous glucose monitoring system in patients with type 2 diabetes on hemodialysis, Ushigome 1570–1574

Hemoglobin

Associations of anemia and hemoglobin with hemoglobin A1c among non‐diabetic workers in Japan, Sakamoto 719–725

Hemoglobin A1c

Associations of anemia and hemoglobin with hemoglobin A1c among non‐diabetic workers in Japan, Sakamoto 719–725
Vitamin D levels, prediabetes risk and hemoglobin A1c levels in young non‐diabetic Saudi women, Al‐Mohaissen 1344–1351

Hemoglobin variant

Silent hemoglobin variant during capillary electrophoresis: A case report, Yuan 1014–1017

Heparin

Insulin and heparin challenge tests are useful for choosing an optimal insulin regimen in a case of subcutaneous insulin resistance, Nakamura 1370–1373

High‐resolution magnetic resonance imaging

Association between type 2 diabetes mellitus, especially recently uncontrolled glycemia, and intracranial plaque characteristics: A high‐resolution magnetic resonance imaging study, Huang 1278–1284

Hip fracture

Impact of hip fracture on all‐cause mortality in Japanese patients with type 2 diabetes mellitus: The Fukuoka Diabetes Registry, Komorita 62–69

Homocysteine

Relationship between metformin use and vitamin B12 status in patients with type 2 diabetes in Japan, Sugawara 917–922

Hospitalization

A territory‐wide study on the impact of COVID‐19 on diabetes‐related acute care, Lui 1303–1306

Human leukocyte antigens

Clinical and genetic characteristics of people with type 1 diabetes who have discrepancies in titers of anti‐glutamic acid decarboxylase antibody measured by radioimmunoassay and enzyme‐linked immunosorbent assay, Takagi 356–362

Hyperinsulinemic hypoglycemia in infancy

Identification of a compound heterozygous inactivating *ABCC8* gene mutation responsible for young‐onset diabetes with exome sequencing, Matsutani 333–336

Hyperinsulinism

Nationwide survey of endogenous hyperinsulinemic hypoglycemia in Japan (2017–2018): Congenital hyperinsulinism, insulinoma, non‐insulinoma pancreatogenous hypoglycemia syndrome and insulin autoimmune syndrome (Hirata’s disease), Yamada 554–563

Hypoglycemia

Recommendations on the Proper Use of SGLT2 Inhibitors, The Committee on the Proper Use of SGLT2 Inhibitors 257–261
Relationship between glycated hemoglobin level and duration of hypoglycemia in type 2 diabetes patients treated with sulfonylureas: A multicenter cross‐sectional study, Matsuoka 417–425
Absolute risk of acute coronary syndrome after severe hypoglycemia: A population‐based 2‐year cohort study using the National Database in Japan, Nishioka 426–434
Nationwide survey of endogenous hyperinsulinemic hypoglycemia in Japan (2017–2018): Congenital hyperinsulinism, insulinoma, non‐insulinoma pancreatogenous hypoglycemia syndrome and insulin autoimmune syndrome (Hirata’s disease), Yamada 554–563
New perspectives on insulin therapy, Araki 795–797
Incidence of severe hypoglycemia and its association with serum adiponectin in Japanese patients with type 1 and insulin‐treated type 2 diabetes: The Fukuoka Diabetes Registry, Iwase 1258–1264

Impaired awareness of hypoglycemia

Hypoglycemia unawareness and autonomic dysfunction in diabetes: Lessons learned and roles of diabetes technologies, Lin 1388–1402

Impaired fasting glucose

Risk and population attributable fraction of metabolic syndrome and impaired fasting glucose for the incidence of type 2 diabetes mellitus among middle‐aged Japanese individuals: Aichi Worker’s Cohort Study, Kaneko 1163–1169

Incident diabetes

Triglyceride to high‐density lipoprotein cholesterol ratio is associated with incident diabetes in men: A retrospective study of Chinese individuals, Qin 192–198

Incretin

Tumor‐like features of gene expression and metabolic profiles in enlarged pancreatic islets are associated with impaired incretin‐induced insulin secretion in obese diabetes: A study of Zucker fatty diabetes mellitus rat, Hayami 1434–1447

Incretins

Factors associated with the glucose‐lowering efficacy of sitagliptin in Japanese patients with type 2 diabetes mellitus: Pooled analysis of Japanese clinical trials, Tajima 640–646

Inositol‐requiring enzyme 1α

Nicotinic acetylcholine receptor signaling regulates inositol‐requiring enzyme 1α activation to protect β‐cells against terminal unfolded protein response under irremediable endoplasmic reticulum stress, Ishibashi 801–813

Inpatients

Dulaglutide‐combined basal plus correction insulin therapy contributes to ideal glycemic control in non‐critical hospitalized patients, Fushimi 125–131

*INS* mutation

Identification of insulin gene variants in patients with neonatal diabetes in the Chinese population, Fu 578–584

Insulin

Long‐term (52‐week) efficacy and safety of ipragliflozin add‐on therapy to insulin in Japanese patients with type 1 diabetes mellitus: An uncontrolled, open‐label extension of a phase III study, Kaku 662–671
Ultra‐Rapid Lispro results in accelerated insulin lispro absorption and faster early insulin action in comparison with Humalog® in Japanese patients with type 1 diabetes, Shiramoto 672–680
Intra‐individual association between C‐reactive protein and insulin administration in postoperative lumbar spinal canal stenosis patients: A retrospective cohort study, Kurisu 980–984
Insulin‐derived amyloidosis without a palpable mass at the insulin injection site: A report of two cases, Nagase 1002–1005
Coping styles associated with glucose control in individuals with type 2 diabetes mellitus, Murakami 1215–1221
Incidence of severe hypoglycemia and its association with serum adiponectin in Japanese patients with type 1 and insulin‐treated type 2 diabetes: The Fukuoka Diabetes Registry, Iwase 1258–1264
Therapeutic potential for insulin on type 1 diabetes‐associated periodontitis: Analysis of experimental periodontitis in streptozotocin‐induced diabetic rats, Nishikawa 1482–1489

Insulin analog

New perspectives on insulin therapy, Araki 795–797

Insulin clearance

Shape of the glucose response curve during an oral glucose tolerance test is associated with insulin clearance and muscle insulin sensitivity in healthy non‐obese men, Kaga 874–877

Insulin‐dependent diabetes mellitus

Long‐term outcome of islet transplantation on insulin‐dependent diabetes mellitus: An observational cohort study, Nakamura 363–372

Insulinogenic index

Characteristics associated with elevated 1‐h plasma glucose levels during a 75‐g oral glucose tolerance test in non‐obese Japanese men, Sato 1520–1523

Insulin pump

Effect of real‐life insulin pump with predictive low‐glucose management use for 3 months: Analysis of the patients treated in a Japanese center, Tsunemi 1564–1569

Insulin resistance

Elevation of the renal threshold for glucose is associated with insulin resistance and higher glycated hemoglobin levels, Hieshima 617–625
Shape of the glucose response curve during an oral glucose tolerance test is associated with insulin clearance and muscle insulin sensitivity in healthy non‐obese men, Kaga 874–877
Association between plasma irisin and glucose metabolism in pregnant women is modified by dietary n‐3 polyunsaturated fatty acid intake, Cai 1326–1335
Low birthweight is associated with type 2 diabetes mellitus in Japanese adults: The Toon Health Study, Yokoyama 1643–1650

Insulin resistance syndrome

Clinical characteristics of insulin resistance syndromes: A nationwide survey in Japan, Takeuchi 603–616

Insulin secretion

Characterization of the taste receptor‐related G‐protein, α‐gustducin, in pancreatic β‐cells, Udagawa 814–822
Alcohol‐induced impaired insulin secretion in a Japanese population: 5‐year follow up in the Gifu Diabetes Study, Ueda 1207–1214

Insulin sensitivity

Apolipoprotein M overexpression through adeno‐associated virus gene transfer improves insulin secretion and insulin sensitivity in Goto‐Kakizaki rats, Yu 1150–1158

Insulin therapy

Effects of liraglutide and empagliflozin added to insulin therapy in patients with type 2 diabetes: A randomized controlled study, Nakaguchi 1542–1550

International Association of Diabetes and Pregnancy Study Groups criteria

Long‐term maternal cardiometabolic outcomes 22 years after gestational diabetes mellitus, Tutino 985–993

Intracranial atherosclerosis

Association between type 2 diabetes mellitus, especially recently uncontrolled glycemia, and intracranial plaque characteristics: A high‐resolution magnetic resonance imaging study, Huang 1278–1284

Invasive fungal disease

Opportunistic invasive fungal disease in patients with type 2 diabetes mellitus from Southern China: Clinical features and associated factors, Lao 731–744

Ipragliflozin

Effects of ipragliflozin on the development and progression of kidney disease in patients with type 2 diabetes: An analysis from a multicenter prospective intervention study, Matsuba 1248–1257
Long‐term efficacy of the sodium–glucose cotransporter 2 inhibitor, ipragliflozin, in a case of type A insulin resistance syndrome, Nagashima 1363–1365

Irisin

Association between plasma irisin and glucose metabolism in pregnant women is modified by dietary n‐3 polyunsaturated fatty acid intake, Cai 1326–1335

Islet fibrosis

Pancreatic stellate cells in the islets as a novel target to preserve the pancreatic β‐cell mass and function, Yang 268–280

Islet stellate cells

Wingless‐type MMTV integration site family member 5a is a key inhibitor of islet stellate cells activation, Xu 307–314

Islet transplantation

Long‐term outcome of islet transplantation on insulin‐dependent diabetes mellitus: An observational cohort study, Nakamura 363–372
Reduced glycemic variability and flexible graft function after islet transplantation: A case report, Nakamura 1677–1680

Insulin secretion capacity

Pancreatic fat is related to the longitudinal decrease in the increment of C‐peptide in glucagon stimulation test in type 2 diabetes patients, Ishibashi 80–87

Intermittently scanned continuous glucose monitoring

Reduced glycemic variability and flexible graft function after islet transplantation: A case report, Nakamura 1677–1680

Japan Diabetes Complication and its Prevention study

Prevalence of albuminuria and renal dysfunction, and related clinical factors in Japanese patients with diabetes: The Japan Diabetes Complication and its Prevention prospective study 5, Shikata 325–332

Japanese

Ultra‐Rapid Lispro results in accelerated insulin lispro absorption and faster early insulin action in comparison with Humalog® in Japanese patients with type 1 diabetes, Shiramoto 672–680
Alcohol‐induced impaired insulin secretion in a Japanese population: 5‐year follow up in the Gifu Diabetes Study, Ueda 1207–1214

JDI Updates

Diabetic polyneuropathy: Progress in diagnostic strategy and novel target discovery, but stagnation in drug development, Himeno 25–27
Obesity, metabolic syndrome and bariatric surgery: A narrative review, Jia 294‐296
New perspective on type 2 diabetes, dyslipidemia and non‐alcoholic fatty liver disease, Matsuzaka 532–534
New perspectives on insulin therapy, Araki 795–797
Clinical evidence regarding factors linking metabolic abnormal obesity to pancreatic β‐cell dysfunction, Watada 798–800
Beginning of a new era in glucagon research: Breakthrough by the new glucagon assay, Kawamori 1123–1125
SGLT2 inhibitors for genetic and acquired insulin resistance: Considerations for clinical use, Hosokawa 1431–1433

Ketoacidosis

Recommendations on the Proper Use of SGLT2 Inhibitors, The Committee on the Proper Use of SGLT2 Inhibitors 257–261

Ketone body

Effect of tofogliflozin on cardiac and vascular endothelial function in patients with type 2 diabetes and heart diseases: A pilot study, Tochiya 400–404

Ketosis

Sodium–glucose cotransporter 2 inhibitor and sarcopenia in a lean elderly adult with type 2 diabetes: A case report, Yasuda 745–747

Ketosis‐prone type 2 diabetes

Bodyweight threshold for sudden onset of ketosis might exist in ketosis‐prone type 2 diabetes patients, Satomura 499–501

Kidney injury molecule‐1

Circulating kidney injury molecule‐1 as a biomarker of renal parameters in diabetic kidney disease, Gohda 435–440

Latent autoimmune diabetes in youth

Juvenile case with the coexistence of maturity‐onset diabetes of the young 1 and later‐onset latent autoimmune diabetes in youth, Yoshida 1010–1013

Left ventricular diastolic function

Effect of tofogliflozin on cardiac and vascular endothelial function in patients with type 2 diabetes and heart diseases: A pilot study, Tochiya 400–404

Leptin

Inverse association of plasma leptin with cortical thickness at distal radius determined with a quantitative ultrasound device in patients with type 2 diabetes mellitus, Kurajoh 174–183

Letter to the Editor

Anti‐programmed death ligand 1 therapy‐induced type 1 diabetes presenting with multiple islet‐related autoantibodies, Honoki 253–254
Caution is required for the evaluation of the accuracy of continuous glucose monitoring devices, Hirota 255
Reply to the comment of Hirot *et al*. on “Accuracy of flash glucose monitoring in insulin‐treated patients with type 2 diabetes”, Sato 256
Facilitating screening of Klinefelter syndrome among patients with diabetes, Seno 506‐507
Role of incretin‐based therapy in hospitalized patients with type 2 diabetes, Gracia‐Ramos 508‐509
Nivolumab‐induced fulminant type 1 diabetes with precipitous fall in C‐peptide level, Miyauchi 748‐749
Levels of fasting plasma glucose in non‐hospitalized older people with high hemoglobin A1c levels, Kashima 750‐751
Integrated 3 in 1 Siriraj Diabetes School Camp: Views, reflections and lessons learned by participating healthcare professionals, Preechasuk 1018‐1019
Homonymous quadrantanopia associated with hyperosmolar hyperglycemic syndrome, Mizuguchi 1374‐1375

Life expectancy

Causes of death and estimated life expectancy among people with diabetes: A retrospective cohort study in a diabetes clinic, Goto 52–54

Lifestyle

A 50‐year history of the health impacts of Westernization on the lifestyle of Japanese Americans: A focus on the Hawaii–Los Angeles–Hiroshima Study, Yoneda 1382–1387

Linear mixed effects model

Intra‐individual association between C‐reactive protein and insulin administration in postoperative lumbar spinal canal stenosis patients: A retrospective cohort study, Kurisu 980–984

Liraglutide

Effects of liraglutide and empagliflozin added to insulin therapy in patients with type 2 diabetes: A randomized controlled study, Nakaguchi 1542–1550

Long‐term outcome

Long‐term outcome of islet transplantation on insulin‐dependent diabetes mellitus: An observational cohort study, Nakamura 363–372

Low‐dose liraglutide

Evaluation of the efficacy of low‐dose liraglutide in weight control among Taiwanese non‐diabetes patients, Chou 1524–1531

Macrophages

Metformin shows anti‐inflammatory effects in murine macrophages through Dicer/microribonucleic acid‐34a‐5p and microribonucleic acid‐125b‐5p, Luo 101–109

Macrovascular complication

Greater macrovascular and microvascular morbidity from type 2 diabetes in northern compared with southern China: A cross‐sectional study, Wang 1285–1294

Magnetic resonance imaging

Elevated serum glycated albumin and glycated albumin : hemoglobin A1c ratio were associated with hippocampal atrophy in a general elderly population of Japanese: The Hisayama Study, Ohara 971–979

Management

Comparison of pregnancy outcomes between women with early‐onset and late‐onset gestational diabetes in a retrospective multi‐institutional study in Japan, Usami 216–222

Maturity‐onset diabetes of the young 1

Juvenile case with the coexistence of maturity‐onset diabetes of the young 1 and later‐onset latent autoimmune diabetes in youth, Yoshida 1010–1013

Mean platelet volume

Association between mean platelet volume in the pathogenesis of type 2 diabetes mellitus and diabetic macrovascular complications in Japanese patients, Inoue 938–945

Medical nutrition therapy

Standard medical nutrition therapy of 25 kcal/kg ideal bodyweight/day often does not reach even resting energy expenditure for patients with type 2 diabetes, Fukuda 626–632

Mendelian randomization

Association between cancer antigen 19‐9 and diabetes risk: A prospective and Mendelian randomization study, Li 585–593

Meta‐analysis

Association between the ferritin level and risk of gestational diabetes mellitus: A meta‐analysis of observational studies, Sun 707–718

Metabolic diseases

Dementia risk by combinations of metabolic diseases and body mass index: Japan Gerontological Evaluation Study Cohort Study, Yokomichi 206–215

Metabolic syndrome

Association between serum 25‐hydroxyvitamin D concentrations and metabolic syndrome in the middle‐aged and elderly Chinese population in Dalian, northeast China: A cross‐sectional study, Weldegiorgis 184–191
Risk and population attributable fraction of metabolic syndrome and impaired fasting glucose for the incidence of type 2 diabetes mellitus among middle‐aged Japanese individuals: Aichi Worker’s Cohort Study, Kaneko 1163–1169
Risk of type 2 diabetes according to the cumulative exposure to metabolic syndrome or obesity: A nationwide population‐based study, Lee 1583–1593

Metformin

Metformin shows anti‐inflammatory effects in murine macrophages through Dicer/microribonucleic acid‐34a‐5p and microribonucleic acid‐125b‐5p, Luo 101–109
Relationship between metformin use and vitamin B_12_ status in patients with type 2 diabetes in Japan, Sugawara 917–922

Microcirculation

Decreased microcirculatory function measured by perfusion index is a novel indicator of diabetic kidney disease in patients with type 2 diabetes, Okada 681–687

Microribonucleic acids

Metformin shows anti‐inflammatory effects in murine macrophages through Dicer/microribonucleic acid‐34a‐5p and microribonucleic acid‐125b‐5p, Luo 101–109

Microvascular complication

Greater macrovascular and microvascular morbidity from type 2 diabetes in northern compared with southern China: A cross‐sectional study, Wang 1285–1294

Mild cognitive impairment

Prevalence of mild cognitive impairment in type 2 diabetes mellitus is associated with serum galectin‐3 level, Ma 1295–1302

Mirogabalin

Long‐term safety and efficacy of mirogabalin in Asian patients with diabetic peripheral neuropathic pain, Baba 693–698

Mixed‐meal test

Reduced glycemic variability and flexible graft function after islet transplantation: A case report, Nakamura 1677–1680

Mortality

Causes of death and estimated life expectancy among people with diabetes: A retrospective cohort study in a diabetes clinic, Goto 52–54
Combined effect of diabetic retinopathy and diabetic kidney disease on all‐cause, cancer, vascular and non‐cancer non‐vascular mortality in patients with type 2 diabetes: A real‐world longitudinal study, Takao 1170–1180

Mortality risk

Cancer has overtaken cardiovascular disease as the commonest cause of death in Scottish type 2 diabetes patients: A population‐based study (The Ayrshire Diabetes Follow‐up Cohort study), Collier 55–61

Multimorbidity

Prevalence of diabetes in Japanese patients with cancer, Saito 1159–1162

Muscle mass

Effects of dapagliflozin on the serum levels of fibroblast growth factor 21 and myokines and muscle mass in Japanese patients with type 2 diabetes: A randomized, controlled trial, Yamakage 653–661

Muscle strength

Handgrip measurement as a useful benchmark for locomotive syndrome in patients with type 2 diabetes mellitus: A KAMOGAWA‐DM cohort study, Kitagawa 1602–1611

Myocardial ischemia‐reperfusion injury

Activation of autophagy inhibits nucleotide‐binding oligomerization domain‐like receptor protein 3 inflammasome activation and attenuates myocardial ischemia‐reperfusion injury in diabetic rats, Zhang 1126–1136

n‐3 polyunsaturated fatty acid

Association between plasma irisin and glucose metabolism in pregnant women is modified by dietary n‐3 polyunsaturated fatty acid intake, Cai 1326–1335

*n*−3 polyunsaturated fatty acids

Ratios of serum eicosapentaenoic acid to arachidonic acid and docosahexaenoic acid to arachidonic acid were inversely associated with serum resistin levels: The Hisayama Study, Higashioka 482–489

N‐acetyl‐seryl‐aspartyl‐lysyl‐proline

CD‐1*^db/db^* mice: A novel type 2 diabetic mouse model with progressive kidney fibrosis, Mizunuma 1470–1481

Nationwide survey

Clinical characteristics of insulin resistance syndromes: A nationwide survey in Japan, Takeuchi 603–616

Neonatal diabetes

Neonatal diabetes caused by the heterozygous Pro1198Leu mutation in the *ABCC8* gene in a male infant: 6‐year clinical course, Uraki 502–505

Neonatal diabetes mellitus

Identification of insulin gene variants in patients with neonatal diabetes in the Chinese population, Fu 578–584

Newly diagnosed

Evaluation of effectiveness of treatment paradigm for newly diagnosed type 2 diabetes patients in China: A nationwide prospective cohort study, Cai 151–161

Nicotinamide

Impaired nicotinamide adenine dinucleotide (NAD^+^) metabolism in diabetes and diabetic tissues: Implications for nicotinamide‐related compound treatment, Fan 1403–1419

Nicotinamide adenine dinucleotide

Impaired nicotinamide adenine dinucleotide (NAD^+^) metabolism in diabetes and diabetic tissues: Implications for nicotinamide‐related compound treatment, Fan 1403–1419

Nicotinic acetylcholine receptor

Nicotinic acetylcholine receptor signaling regulates inositol‐requiring enzyme 1α activation to protect β‐cells against terminal unfolded protein response under irremediable endoplasmic reticulum stress, Ishibashi 801–813

Non‐alcoholic fatty liver disease

Effects of sodium–glucose cotransporter 2 inhibitors on non‐alcoholic fatty liver disease in patients with type 2 diabetes: A meta‐analysis of randomized controlled trials, Xing 1238–1247
Increased risks of different grades of non‐alcoholic fatty liver disease in prediabetic subjects with impaired fasting glucose and glucose tolerance, including the isolated glycosylated hemoglobin levels of 5.7–6.4% in a Chinese population, Li 1336–1343
Comparison of the effects of three kinds of glucose‐lowering drugs on non‐alcoholic fatty liver disease in patients with type 2 diabetes: A randomized, open‐label, three‐arm, active control study, Kinoshita 1612–1622

Non‐invasive

Three‐component non‐invasive risk score for undiagnosed diabetes in Chinese people: Development, validation and longitudinal evaluation, Woo 341–348

Non‐linear model

Factors associated with diabetic polyneuropathy‐related sensory symptoms and signs in patients with polyneuropathy: A cross‐sectional Japanese study (JDDM 52) using a non‐linear model, Yokoyama 450–457

Non‐obese

Characteristics associated with elevated 1‐h plasma glucose levels during a 75‐g oral glucose tolerance test in non‐obese Japanese men, Sato 1520–1523

Nonvolatile acid

Low urine pH predicts new onset of diabetes mellitus during a 10‐year period in men: BOREAS‐DM1 study, Higashiura 1490–1497
Nucleotide‐binding oligomerization domain‐like receptor protein 3 inflammasome
Activation of autophagy inhibits nucleotide‐binding oligomerization domain‐like receptor protein 3 inflammasome activation and attenuates myocardial ischemia‐reperfusion injury in diabetic rats, Zhang 1126–1136

Obesity

Longitudinal examination of pancreatic β‐cell function in Japanese individuals, Fujikawa 70–74
Inverse association of plasma leptin with cortical thickness at distal radius determined with a quantitative ultrasound device in patients with type 2 diabetes mellitus, Kurajoh 174–183
Bodyweight threshold for sudden onset of ketosis might exist in ketosis‐prone type 2 diabetes patients, Satomura 499–501
Long‐term maternal cardiometabolic outcomes 22 years after gestational diabetes mellitus, Tutino 985–993
A 50‐year history of the health impacts of Westernization on the lifestyle of Japanese Americans: A focus on the Hawaii–Los Angeles–Hiroshima Study, Yoneda 1382–1387
Safflower yellow improves insulin sensitivity in high‐fat diet‐induced obese mice by promoting peroxisome proliferator‐activated receptor‐γ2 expression in subcutaneous adipose tissue, Yan 1457–1469
Different pathogenesis of glucose intolerance in two subtypes of primary aldosteronism: Aldosterone‐producing adenoma and idiopathic hyperaldosteronism, Okazaki‐Hada 1511–1519
Risk of type 2 diabetes according to the cumulative exposure to metabolic syndrome or obesity: A nationwide population‐based study, Lee 1583–1593

Oral antidiabetic drugs

Comparing treatment intensification and clinical outcomes of metformin and dipeptidyl peptidase‐4 inhibitors in treatment‐naïve patients with type 2 diabetes in Japan, Horii 96–100

Oral glucose tolerance test

Longitudinal examination of pancreatic β‐cell function in Japanese individuals, Fujikawa 70–74

Osteoporosis

Association between the Geriatric Nutritional Risk Index, bone mineral density and osteoporosis in type 2 diabetes patients, Wang 956–963

Pain

Long‐term safety and efficacy of mirogabalin in Asian patients with diabetic peripheral neuropathic pain, Baba 693–698

Pancreatic β cell

Nicotinic acetylcholine receptor signaling regulates inositol‐requiring enzyme 1α activation to protect β‐cells against terminal unfolded protein response under irremediable endoplasmic reticulum stress, Ishibashi 801–813

Pancreatic β‐cell dysfunction

Correlation between serum proinsulin levels and fatty liver: The Dynamics of Lifestyle and Neighborhood Community on Health Study Health Study, Miya 964–970

Pancreatic fat

Pancreatic fat is related to the longitudinal decrease in the increment of C‐peptide in glucagon stimulation test in type 2 diabetes patients, Ishibashi 80–87

Pancreatic fat volume

Pancreatic fat accumulation evaluated by multidetector computed tomography in patients with type 2 diabetes, Fukase 1188–1196

Pancreatic islet

Adrenomedullin has a cytoprotective role against endoplasmic reticulum stress for pancreatic β‐cells in autocrine and paracrine manners, Suetomi 823–833

Pancreatic stellate cells

Pancreatic stellate cells in the islets as a novel target to preserve the pancreatic β‐cell mass and function, Yang 268–280

Passive body trunk exercise equipment

Effect of the use of passive body trunk exercise equipment on oxygen consumption and self‐efficacy for carrying out exercise in patients with type 2 diabetes, Kawae 1265–1271

Perfusion index

Decreased microcirculatory function measured by perfusion index is a novel indicator of diabetic kidney disease in patients with type 2 diabetes, Okada 681–687

Periodontitis

Therapeutic potential for insulin on type 1 diabetes‐associated periodontitis: Analysis of experimental periodontitis in streptozotocin‐induced diabetic rats, Nishikawa 1482–1489

Peripheral neuropathy

Diagnosing peripheral neuropathy in South‐East Asia: A focus on diabetic neuropathy, Malik 1097–1103

Peroxisome proliferator‐activated receptor gamma

Association between Pro12Ala polymorphism and albuminuria in type 2 diabetic nephropathy, Chen 923–929

Peroxisome proliferator‐activated receptor‐γ2

Safflower yellow improves insulin sensitivity in high‐fat diet‐induced obese mice by promoting peroxisome proliferator‐activated receptor‐γ2 expression in subcutaneous adipose tissue, Yan 1457–1469

Phosphatidylinositol 3‐kinase–protein kinase B signaling pathway

Berberine ameliorates renal impairment and inhibits podocyte dysfunction by targeting the phosphatidylinositol 3‐kinase–protein kinase B pathway in diabetic rats, Ni 297–306

Physical activity

Impact of physical activity and sedentary time on glycated hemoglobin levels and body composition: Cross‐sectional study using outpatient clinical data of Japanese patients with type 2 diabetes, Nakanishi 633–639

Placenta

Whole transcriptome expression profiles in placenta samples from women with gestational diabetes mellitus, Tang 1307–1317

Podocyte dysfunction

Berberine ameliorates renal impairment and inhibits podocyte dysfunction by targeting the phosphatidylinositol 3‐kinase–protein kinase B pathway in diabetic rats, Ni 297–306

Polymorphism

Association between Pro12Ala polymorphism and albuminuria in type 2 diabetic nephropathy, Chen 923–929

Polyneuropathy

Polyneuropathy is inadequately treated despite increasing symptom intensity in individuals with and without diabetes (PROTECT follow‐up study), Ziegler 1272–1277

Population attributable fraction and type 2 diabetes mellitus

Risk and population attributable fraction of metabolic syndrome and impaired fasting glucose for the incidence of type 2 diabetes mellitus among middle‐aged Japanese individuals: Aichi Worker’s Cohort Study, Kaneko 1163–1169

Post‐marketing study

Safety and effectiveness of tofogliflozin in Japanese patients with type 2 diabetes mellitus in real‐world practice: Results of 12‐month interim analysis of a long‐term post‐marketing surveillance study (J‐STEP/LT), Utsunomiya 132–141
Safety and effectiveness of tofogliflozin in Japanese patients with type 2 diabetes mellitus: Results of 24‐month interim analysis of a long‐term post‐marketing study (J‐STEP/LT), Utsunomiya 906–916

Postmenopausal

Increasing risk of diabetes mellitus in postmenopausal women with newly diagnosed primary breast cancer, Wang 490–498

Postprandial hyperglycemia

Effect of postprandial hyperglycemia at clinic visits on the incidence of retinopathy in patients with type 2 diabetes: An analysis using real‐world long‐term follow‐up data, Takao 930–937

Post‐suspend hyperglycemia

Effect of real‐life insulin pump with predictive low‐glucose management use for 3 months: Analysis of the patients treated in a Japanese center, Tsunemi 1564–1569

Prediabetes

Association of glucose tolerance status with pancreatic β‐ and α‐cell mass in community‐based autopsy samples of Japanese individuals: The Hisayama Study, Inaishi 1197–1206
Increased risks of different grades of non‐alcoholic fatty liver disease in prediabetic subjects with impaired fasting glucose and glucose tolerance, including the isolated glycosylated hemoglobin levels of 5.7–6.4% in a Chinese population, Li 1336–1343
Vitamin D levels, prediabetes risk and hemoglobin A1c levels in young non‐diabetic Saudi women, Al‐Mohaissen 1344–1351

Prediabetic state

Early sensory neurophysiological changes in prediabetes, Lin 458–465

Predictive low‐glucose management

Effect of real‐life insulin pump with predictive low‐glucose management use for 3 months: Analysis of the patients treated in a Japanese center, Tsunemi 1564–1569

Pregnancy

Association between gut microbiota composition and glycoalbumin level during pregnancy in Japanese women: Pilot study from Chiba Study of Mother and Child Health, Sakurai 699–706
Time course of metabolic status in pregnant women: The Japan Environment and Children’s Study, Sasaki 1318–1325
High serum triglyceride levels in the early first trimester of pregnancy are associated with gestational diabetes mellitus: A prospective cohort study, Zhu 1635–1642

Pregnancy outcome

Comparison of pregnancy outcomes between women with early‐onset and late‐onset gestational diabetes in a retrospective multi‐institutional study in Japan, Usami 216–222
Long‐term maternal cardiometabolic outcomes 22 years after gestational diabetes mellitus, Tutino 985–993

Primary aldosteronism

Different pathogenesis of glucose intolerance in two subtypes of primary aldosteronism: Aldosterone‐producing adenoma and idiopathic hyperaldosteronism, Okazaki‐Hada 1511–1519

Proinsulin

Proinsulin is sensitive to reflect glucose intolerance, Nakamura 75–79
Correlation between serum proinsulin levels and fatty liver: The Dynamics of Lifestyle and Neighborhood Community on Health Study Health Study, Miya 964–970

Prostate cancer

Activation of overexpressed glucagon‐like peptide‐1 receptor attenuates prostate cancer growth by inhibiting cell cycle progression, Shigeoka 1137–1149

Protective factor

Protective factors as biomarkers and targets for prevention and treatment of diabetic nephropathy: From current human evidence to future possibilities, Nowak 1085–1096

Purine metabolism

Differential regulation of hypoxanthine and xanthine by obesity in a general population, Furuhashi 878–887

Radioimmunoassay

Increased diagnosis of autoimmune childhood‐onset Japanese type 1 diabetes using a new glutamic acid decarboxylase antibody enzyme‐linked immunosorbent assay kit, compared with a previously used glutamic acid decarboxylase antibody radioimmunoassay kit, Sugihara 594–602

Renal anemia

Failure to confirm a sodium–glucose cotransporter 2 inhibitor‐induced hematopoietic effect in non‐diabetic rats with renal anemia, Yamazaki 834–843

Renal injury

Transplantation of adipose‐derived mesenchymal stem cell sheets directly into the kidney suppresses the progression of renal injury in a diabetic nephropathy rat model, Takemura 545–553

Renal reabsorption

Elevation of the renal threshold for glucose is associated with insulin resistance and higher glycated hemoglobin levels, Hieshima 617–625

Renin–angiotensin system

Pancreatic stellate cells in the islets as a novel target to preserve the pancreatic β‐cell mass and function, Yang 268–280

Resistin

Ratios of serum eicosapentaenoic acid to arachidonic acid and docosahexaenoic acid to arachidonic acid were inversely associated with serum resistin levels: The Hisayama Study, Higashioka 482–489

Resting energy expenditure

Standard medical nutrition therapy of 25 kcal/kg ideal bodyweight/day often does not reach even resting energy expenditure for patients with type 2 diabetes, Fukuda 626–632

Risk factors in epidemiology

Elevated serum glycated albumin and glycated albumin : hemoglobin A1c ratio were associated with hippocampal atrophy in a general elderly population of Japanese: The Hisayama Study, Ohara 971–979

Safflower yellow

Safflower yellow improves insulin sensitivity in high‐fat diet‐induced obese mice by promoting peroxisome proliferator‐activated receptor‐γ2 expression in subcutaneous adipose tissue, Yan 1457–1469

Salvage pathway

Differential regulation of hypoxanthine and xanthine by obesity in a general population, Furuhashi 878–887

Sandwich enzyme‐linked immunosorbent assay

Impaired early‐phase suppression of glucagon secretion after glucose load is associated with insulin requirement during pregnancy in gestational diabetes, Horie 232–240

Sarcopenia

Sodium–glucose cotransporter 2 inhibitor and sarcopenia in a lean elderly adult with type 2 diabetes: A case report, Yasuda 745–747
Handgrip measurement as a useful benchmark for locomotive syndrome in patients with type 2 diabetes mellitus: A KAMOGAWA‐DM cohort study, Kitagawa 1602–1611

SARS‐CoV‐2

Dissecting the interaction between COVID‐19 and diabetes mellitus, Chee 1104–1114

Saudi population

Dysglycemia risk score in Saudi Arabia: A tool to identify people at high future risk of developing type 2 diabetes, Bahijri 844–855

Saxagliptin

Impact of baseline characteristics on glycemic effects of add‐on saxagliptin or acarbose to metformin therapy: Subgroup analysis of the SMART study in Chinese patients with type 2 diabetes mellitus, Fang 896–905

Score

Three‐component non‐invasive risk score for undiagnosed diabetes in Chinese people: Development, validation and longitudinal evaluation, Woo 341–348

Self‐monitoring blood glucose

Flash glucose monitoring in type 1 diabetes: A comparison with self‐monitoring blood glucose, Babaya 1222–1229

Sensory nerve excitability

Early sensory neurophysiological changes in prediabetes, Lin 458–465

Serum glycoalbumin

Association between gut microbiota composition and glycoalbumin level during pregnancy in Japanese women: Pilot study from Chiba Study of Mother and Child Health, Sakurai 699–706

Shape of glucose response curve

Shape of the glucose response curve during an oral glucose tolerance test is associated with insulin clearance and muscle insulin sensitivity in healthy non‐obese men, Kaga 874–877

Sitagliptin

Factors associated with the glucose‐lowering efficacy of sitagliptin in Japanese patients with type 2 diabetes mellitus: Pooled analysis of Japanese clinical trials, Tajima 640–646
Efficacy and safety of metformin and sitagliptin‐based dual and triple therapy in elderly Chinese patients with type 2 diabetes: Subgroup analysis of STRATEGY study, Liu 1532–1541

Skeletal muscle

Handgrip measurement as a useful benchmark for locomotive syndrome in patients with type 2 diabetes mellitus: A KAMOGAWA‐DM cohort study, Kitagawa 1602–1611

Slowly‐progressive type 1 diabetes

Characterization of patients with diabetes who were incidentally found to be glutamic acid decarboxylase autoantibody‐positive by bridging‐type enzyme‐linked immunosorbent assay, Kawasaki 1507–1510

Small fiber neuropathy

Corneal nerve loss in children with type 1 diabetes mellitus without retinopathy or microalbuminuria, Gad 1594–1601

Small‐for‐gestational age

Case of type 2 diabetes possibly caused by excessive accumulation of visceral fat in a child born small‐for‐gestational age, Kuwabara 1366–1369

Sociodemographic

Association between environmental quality and diabetes in the USA, Jagai 315–324

Sodium–glucose cotransporter 1

Sodium–glucose cotransporters: Functional properties and pharmaceutical potential, Sano 770–782

Sodium–glucose cotransporter 2

Sodium–glucose cotransporters: Functional properties and pharmaceutical potential, Sano 770–782

Sodium–glucose cotransporter 2 inhibitor

Circulating complement‐1q tumor necrosis factor‐α‐related protein isoform 5 levels are low in type 2 diabetes patients and reduced by dapagliflozin, Zhang 88–95
Effect of tofogliflozin on cardiac and vascular endothelial function in patients with type 2 diabetes and heart diseases: A pilot study, Tochiya 400–404
Effects of dapagliflozin on the serum levels of fibroblast growth factor 21 and myokines and muscle mass in Japanese patients with type 2 diabetes: A randomized, controlled trial, Yamakage 653–661
Sodium–glucose cotransporter 2 inhibitor and sarcopenia in a lean elderly adult with type 2 diabetes: A case report, Yasuda 745–747
Failure to confirm a sodium–glucose cotransporter 2 inhibitor‐induced hematopoietic effect in non‐diabetic rats with renal anemia, Yamazaki 834–843
Effects of ipragliflozin on the development and progression of kidney disease in patients with type 2 diabetes: An analysis from a multicenter prospective intervention study, Matsuba 1248–1257
Case report of superior mesenteric artery syndrome that developed in a lean type 2 diabetes patient and was associated with rapid body weight loss after sodium–glucose cotransporter 2 inhibitor administration, Hirai 1359–1362
Long‐term efficacy of the sodium–glucose cotransporter 2 inhibitor, ipragliflozin, in a case of type A insulin resistance syndrome, Nagashima 1363–1365
Comparison of the effects of three kinds of glucose‐lowering drugs on non‐alcoholic fatty liver disease in patients with type 2 diabetes: A randomized, open‐label, three‐arm, active control study, Kinoshita 1612–1622

Sodium–glucose cotransporter 2 inhibitors

Long‐term (52‐week) efficacy and safety of ipragliflozin add‐on therapy to insulin in Japanese patients with type 1 diabetes mellitus: An uncontrolled, open‐label extension of a phase III study, Kaku 662–671
Sodium–glucose cotransporter 2 inhibitors improved time‐in‐range without increasing hypoglycemia in Japanese patients with type 1 diabetes: A retrospective, single‐center, pilot study, Suzuki 1230–1237
Effects of sodium–glucose cotransporter 2 inhibitors on non‐alcoholic fatty liver disease in patients with type 2 diabetes: A meta‐analysis of randomized controlled trials, Xing 1238–1247

Sodium–glucose transporter 2

Safety and effectiveness of tofogliflozin in elderly Japanese patients with type 2 diabetes mellitus: A subanalysis of a post‐marketing study (J‐STEP/EL Study), Kaku 405–416
Family history of diabetes in both parents is strongly associated with impaired residual β‐cell function in Japanese type 2 diabetes patients, Iwata 564–572
Safety and effectiveness of tofogliflozin in Japanese patients with type 2 diabetes mellitus: Results of 24‐month interim analysis of a long‐term post‐marketing study (J‐STEP/LT), Utsunomiya 906–916

Soluble transferrin receptor

Iron metabolism and type 2 diabetes mellitus: A meta‐analysis and systematic review, Liu 946–955

Solute carrier family 5 member 2

Family history of diabetes in both parents is strongly associated with impaired residual β‐cell function in Japanese type 2 diabetes patients, Iwata 564–572

South‐East Asia

Diagnosing peripheral neuropathy in South‐East Asia: A focus on diabetic neuropathy, Malik 1097–1103

Sperm deoxyribonucleic acid methylation

Whole genome bisulfite sequencing of human spermatozoa reveals differentially methylated patterns from type 2 diabetic patients, Chen 856–864

Statins

Statin‐induced autoimmune hepatitis in patients with type 1 diabetes: A report of two cases and literature review, Kawasaki 1673–1676

Stem cells from human exfoliated deciduous teeth

Secreted factors from cultured dental pulp stem cells promoted neurite outgrowth of dorsal root ganglion neurons and ameliorated neural functions in streptozotocin‐induced diabetic mice, Miura‐Yura 28–38

Stimulated by retinoic acid 6

Electronegative low‐density lipoprotein of patients with metabolic syndrome induces pathogenesis of aorta through disruption of the stimulated by retinoic acid 6 cascade, Chen 535–544

ST‐segment elevation myocardial infarction

General glycosylated hemoglobin goals potentially increase myocardial infarction severity in diabetes patients with comorbidities: Insights from a nationwide multicenter study, Che 1498–1506

Subcutaneous injections

Insulin‐derived amyloidosis without a palpable mass at the insulin injection site: A report of two cases, Nagase 1002–1005

Subcutaneous insulin resistance

Insulin and heparin challenge tests are useful for choosing an optimal insulin regimen in a case of subcutaneous insulin resistance, Nakamura 1370–1373

Sulfonylurea receptor 1

Neonatal diabetes caused by the heterozygous Pro1198Leu mutation in the *ABCC8* gene in a male infant: 6‐year clinical course, Uraki 502–505

Sulfonylureas

Relationship between glycated hemoglobin level and duration of hypoglycemia in type 2 diabetes patients treated with sulfonylureas: A multicenter cross‐sectional study, Matsuoka 417–425

Superior mesenteric artery syndrome

Case report of superior mesenteric artery syndrome that developed in a lean type 2 diabetes patient and was associated with rapid body weight loss after sodium–glucose cotransporter 2 inhibitor administration, Hirai 1359–1362

Surveys

Nationwide survey of endogenous hyperinsulinemic hypoglycemia in Japan (2017–2018): Congenital hyperinsulinism, insulinoma, non‐insulinoma pancreatogenous hypoglycemia syndrome and insulin autoimmune syndrome (Hirata’s disease), Yamada 554–563

Taiwan

Evaluation of the efficacy of low‐dose liraglutide in weight control among Taiwanese non‐diabetes patients, Chou 1524–1531

Tandem mass spectrometry

Analysis of urinary sphingolipids using liquid chromatography‐tandem mass spectrometry in diabetic nephropathy, Morita 441–449

Taste receptor

Characterization of the taste receptor‐related G‐protein, α‐gustducin, in pancreatic β‐cells, Udagawa 814–822

Time‐in‐range

Sodium–glucose cotransporter 2 inhibitors improved time‐in‐range without increasing hypoglycemia in Japanese patients with type 1 diabetes: A retrospective, single‐center, pilot study, Suzuki 1230–1237

Tobacco smoke pollution

Passive smoking and type 2 diabetes among never‐smoking women: The Japan Public Health Center‐based Prospective Study, Oba 1352–1358

Tofogliflozin

Safety and effectiveness of tofogliflozin in Japanese patients with type 2 diabetes mellitus in real‐world practice: Results of 12‐month interim analysis of a long‐term post‐marketing surveillance study (J‐STEP/LT), Utsunomiya 132–141
Safety and effectiveness of tofogliflozin in Japanese patients with type 2 diabetes mellitus: Results of 24‐month interim analysis of a long‐term post‐marketing study (J‐STEP/LT), Utsunomiya 906–916

Total testosterone

What is the best biological parameter to predict erectile dysfunction in men aged >55 years with type 2 diabetes?, Raharinavalona 170–173

Trajectory

Distinct developmental trajectories of body mass index and diabetes risk: A 5‐year longitudinal study of Chinese adults, Dai 466–474

Transient receptor potential vanilloid 4

Transient receptor potential vanilloid 4 channels as therapeutic targets in diabetes and diabetes‐related complications, Hu 757–769

Treatment

Japanese Clinical Practice Guideline for Diabetes 2019, Arak 1020–1076
Polyneuropathy is inadequately treated despite increasing symptom intensity in individuals with and without diabetes (PROTECT follow‐up study), Ziegler 1272–1277

Treatment intensification

Comparing treatment intensification and clinical outcomes of metformin and dipeptidyl peptidase‐4 inhibitors in treatment‐naïve patients with type 2 diabetes in Japan, Horii 96–100

Treatment satisfaction

Efficacy and safety of once‐weekly exenatide after switching from twice‐daily exenatide in patients with type 2 diabetes, Watanabe 382–388

Trelagliptin

Efficacy and safety of trelagliptin in Japanese patients with type 2 diabetes with severe renal impairment or end‐stage renal disease: Results from a randomized, phase 3 study, Kaku 373–381

Trends

Trends in the prevalence, awareness, treatment and control of diabetes in rural areas of northern China from 1992 to 2011, Zhang 241–249

Triglyceride

High serum triglyceride levels in the early first trimester of pregnancy are associated with gestational diabetes mellitus: A prospective cohort study, Zhu 1635–1642

Triglyceride : high‐density lipoprotein cholesterol ratio

Triglyceride to high‐density lipoprotein cholesterol ratio is associated with incident diabetes in men: A retrospective study of Chinese individuals, Qin 192–198

Triglyceride to high‐density lipoprotein cholesterol ratio

Higher triglyceride to high‐density lipoprotein cholesterol ratio increases cardiovascular risk: 10‐year prospective study in a cohort of Chinese adults, Chen 475–481

Tumor cells

Tumor‐like features of gene expression and metabolic profiles in enlarged pancreatic islets are associated with impaired incretin‐induced insulin secretion in obese diabetes: A study of Zucker fatty diabetes mellitus rat, Hayami 1434–1447

Type 1 diabetes

Consistency of plasma glucagon levels in patients with type 1 diabetes after a 1‐year period, Kawamori 337–340
Clinical and genetic characteristics of people with type 1 diabetes who have discrepancies in titers of anti‐glutamic acid decarboxylase antibody measured by radioimmunoassay and enzyme‐linked immunosorbent assay, Takagi 356–362
Simultaneous development of Graves’ disease and type 1 diabetes during anti‐programmed cell death‐1 therapy: A case report, Kurihara 1006–1009
Zinc transporter 8 autoantibodies complement glutamic acid decarboxylase and insulinoma‐associated antigen‐2 autoantibodies in the identification and characterization of Japanese type 1 diabetes, Kawasaki 1181–1187
Flash glucose monitoring in type 1 diabetes: A comparison with self‐monitoring blood glucose, Babaya 1222–1229
Sodium–glucose cotransporter 2 inhibitors improved time‐in‐range without increasing hypoglycemia in Japanese patients with type 1 diabetes: A retrospective, single‐center, pilot study, Suzuki 1230–1237
Insulin and heparin challenge tests are useful for choosing an optimal insulin regimen in a case of subcutaneous insulin resistance, Nakamura 1370–1373
Hypoglycemia unawareness and autonomic dysfunction in diabetes: Lessons learned and roles of diabetes technologies, Lin 1388–1402
Therapeutic potential for insulin on type 1 diabetes‐associated periodontitis: Analysis of experimental periodontitis in streptozotocin‐induced diabetic rats, Nishikawa 1482–1489
Factors associated with glycemic control in type 1 diabetes patients in China: A cross‐sectional study, Huo 1575–1582
Statin‐induced autoimmune hepatitis in patients with type 1 diabetes: A report of two cases and literature review, Kawasaki 1673–1676

Type 1 diabetes mellitus

Long‐term (52‐week) efficacy and safety of ipragliflozin add‐on therapy to insulin in Japanese patients with type 1 diabetes mellitus: An uncontrolled, open‐label extension of a phase III study, Kaku 662–671
Ultra‐Rapid Lispro results in accelerated insulin lispro absorption and faster early insulin action in comparison with Humalog® in Japanese patients with type 1 diabetes, Shiramoto 672–680
Corneal nerve loss in children with type 1 diabetes mellitus without retinopathy or microalbuminuria, Gad 1594–1601

Type 2 diabetes

Cancer has overtaken cardiovascular disease as the commonest cause of death in Scottish type 2 diabetes patients: A population‐based study (The Ayrshire Diabetes Follow‐up Cohort study), Collier 55–61
Impact of hip fracture on all‐cause mortality in Japanese patients with type 2 diabetes mellitus: The Fukuoka Diabetes Registry, Komorita 62–69
Pancreatic fat is related to the longitudinal decrease in the increment of C‐peptide in glucagon stimulation test in type 2 diabetes patients, Ishibashi 80–87
Dulaglutide‐combined basal plus correction insulin therapy contributes to ideal glycemic control in non‐critical hospitalized patients, Fushimi 125–131
Evaluation of effectiveness of treatment paradigm for newly diagnosed type 2 diabetes patients in China: A nationwide prospective cohort study, Cai 151–161
Association between age at first childbirth and type 2 diabetes in Chinese women, Qu 223–231
Efficacy and safety of once‐weekly exenatide after switching from twice‐daily exenatide in patients with type 2 diabetes, Watanabe 382–388
Family history of diabetes in both parents is strongly associated with impaired residual β‐cell function in Japanese type 2 diabetes patients, Iwata 564–572
Achieving the composite end‐point of glycated hemoglobin <7.0% without weight gain or hypoglycemia with once‐weekly dulaglutide in Chinese patients with type 2 diabetes: A post‐hoc analysis, Xiao 647–652
Effects of dapagliflozin on the serum levels of fibroblast growth factor 21 and myokines and muscle mass in Japanese patients with type 2 diabetes: A randomized, controlled trial, Yamakage 653–661
Impact of baseline characteristics on glycemic effects of add‐on saxagliptin or acarbose to metformin therapy: Subgroup analysis of the SMART study in Chinese patients with type 2 diabetes mellitus, Fang 896–905
Effect of postprandial hyperglycemia at clinic visits on the incidence of retinopathy in patients with type 2 diabetes: An analysis using real‐world long‐term follow‐up data, Takao 930–937
Association between mean platelet volume in the pathogenesis of type 2 diabetes mellitus and diabetic macrovascular complications in Japanese patients, Inoue 938–945
Iron metabolism and type 2 diabetes mellitus: A meta‐analysis and systematic review, Liu 946–955
Long‐term maternal cardiometabolic outcomes 22 years after gestational diabetes mellitus, Tutino 985–993
Zinc transporter 8 autoantibodies complement glutamic acid decarboxylase and insulinoma‐associated antigen‐2 autoantibodies in the identification and characterization of Japanese type 1 diabetes, Kawasaki 1181–1187
Pancreatic fat accumulation evaluated by multidetector computed tomography in patients with type 2 diabetes, Fukase 1188–1196
Effect of the use of passive body trunk exercise equipment on oxygen consumption and self‐efficacy for carrying out exercise in patients with type 2 diabetes, Kawae 1265–1271
Polyneuropathy is inadequately treated despite increasing symptom intensity in individuals with and without diabetes (PROTECT follow‐up study), Ziegler 1272–1277
Greater macrovascular and microvascular morbidity from type 2 diabetes in northern compared with southern China: A cross‐sectional study, Wang 1285–1294
Prevalence of mild cognitive impairment in type 2 diabetes mellitus is associated with serum galectin‐3 level, Ma 1295–1302
Case of type 2 diabetes possibly caused by excessive accumulation of visceral fat in a child born small‐for‐gestational age, Kuwabara 1366–1369
Intake of sucrose affects gut dysbiosis in patients with type 2 diabetes, Hashimoto, 1623–1634

Type 2 diabetes mellitus

Circulating complement‐1q tumor necrosis factor‐α‐related protein isoform 5 levels are low in type 2 diabetes patients and reduced by dapagliflozin, Zhang 88–95
Comparing treatment intensification and clinical outcomes of metformin and dipeptidyl peptidase‐4 inhibitors in treatment‐naïve patients with type 2 diabetes in Japan, Horii 96–100
Cardiovascular outcomes of vildagliptin in patients with type 2 diabetes mellitus after acute coronary syndrome or acute ischemic stroke, Chen 110–124
Safety and effectiveness of tofogliflozin in Japanese patients with type 2 diabetes mellitus in real‐world practice: Results of 12‐month interim analysis of a long‐term post‐marketing surveillance study (J‐STEP/LT), Utsunomiya 132–141
Efficacy and safety of dulaglutide monotherapy compared with glimepiride in Chinese patients with type 2 diabetes: Post‐hoc analyses of a randomized, double‐blind, phase III study, Shi 142–150
Wingless‐type MMTV integration site family member 5a is a key inhibitor of islet stellate cells activation, Xu 307–314
Increased serum level and impaired response to glucose fluctuation of asprosin is associated with type 2 diabetes mellitus, Zhang 349–355
Efficacy and safety of trelagliptin in Japanese patients with type 2 diabetes with severe renal impairment or end‐stage renal disease: Results from a randomized, phase 3 study, Kaku 373–381
Safety and effectiveness of tofogliflozin in elderly Japanese patients with type 2 diabetes mellitus: A subanalysis of a post‐marketing study (J‐STEP/EL Study), Kaku 405–416
Factors associated with diabetic polyneuropathy‐related sensory symptoms and signs in patients with polyneuropathy: A cross‐sectional Japanese study (JDDM 52) using a non‐linear model, Yokoyama 450–457
Whole genome bisulfite sequencing of human spermatozoa reveals differentially methylated patterns from type 2 diabetic patients, Chen 856–864
Association of glucose tolerance status with pancreatic β‐ and α‐cell mass in community‐based autopsy samples of Japanese individuals: The Hisayama Study, Inaishi 1197–1206
Effects of sodium–glucose cotransporter 2 inhibitors on non‐alcoholic fatty liver disease in patients with type 2 diabetes: A meta‐analysis of randomized controlled trials, Xing 1238‐1247
Efficacy and safety of metformin and sitagliptin‐based dual and triple therapy in elderly Chinese patients with type 2 diabetes: Subgroup analysis of STRATEGY study, Liu 1532–1541
Risk of type 2 diabetes according to the cumulative exposure to metabolic syndrome or obesity: A nationwide population‐based study, Lee 1583–1593
Comparison of the effects of three kinds of glucose‐lowering drugs on non‐alcoholic fatty liver disease in patients with type 2 diabetes: A randomized, open‐label, three‐arm, active control study, Kinoshita 1612–1622
Low birthweight is associated with type 2 diabetes mellitus in Japanese adults: The Toon Health Study, Yokoyama 1643–1650
Healthcare utilization and direct medical cost in the years during and after cancer diagnosis in patients with type 2 diabetes mellitus, Wu 1661–1672

Type A insulin resistance syndrome

Long‐term efficacy of the sodium–glucose cotransporter 2 inhibitor, ipragliflozin, in a case of type A insulin resistance syndrome, Nagashima 1363–1365

Underweight

Dementia risk by combinations of metabolic diseases and body mass index: Japan Gerontological Evaluation Study Cohort Study, Yokomichi 206–215

Urine

Analysis of urinary sphingolipids using liquid chromatography‐tandem mass spectrometry in diabetic nephropathy, Morita 441–449

Urine pH

Low urine pH predicts new onset of diabetes mellitus during a 10‐year period in men: BOREAS‐DM1 study, Higashiura 1490–1497

Vildagliptin

Cardiovascular outcomes of vildagliptin in patients with type 2 diabetes mellitus after acute coronary syndrome or acute ischemic stroke, Chen 110–124

Vitamin D

Association between serum 25‐hydroxyvitamin D concentrations and metabolic syndrome in the middle‐aged and elderly Chinese population in Dalian, northeast China: A cross‐sectional study, Weldegiorgis 184–191
Vitamin D levels, prediabetes risk and hemoglobin A1c levels in young non‐diabetic Saudi women, Al‐Mohaissen 1344–1351

Visceral fat

Is visceral abdominal fat area a better indicator for hyperglycemic risk? Results from the Pinggu Metabolic Disease Study, Zhang 888–895
Case of type 2 diabetes possibly caused by excessive accumulation of visceral fat in a child born small‐for‐gestational age, Kuwabara 1366–1369

Vitamin B_12_

Relationship between metformin use and vitamin B_12_ status in patients with type 2 diabetes in Japan, Sugawara 917–922

Vitreous hemorrhage

Vitreous hemorrhage in diabetes patients with proliferative diabetic retinopathy undergoing hemodialysis, Kameda 688–692

Weight reduction

Evaluation of the efficacy of low‐dose liraglutide in weight control among Taiwanese non‐diabetes patients, Chou 1524–1531

Whole‐genome bisulfite sequencing

Whole genome bisulfite sequencing of human spermatozoa reveals differentially methylated patterns from type 2 diabetic patients, Chen 856–864

Whole transcriptome

Whole transcriptome expression profiles in placenta samples from women with gestational diabetes mellitus, Tang 1307–1317
Wingless‐type MMTV integration site family member 5a
Wingless‐type MMTV integration site family member 5a is a key inhibitor of islet stellate cells activation, Xu 307–314

Women

Passive smoking and type 2 diabetes among never‐smoking women: The Japan Public Health Center‐based Prospective Study, Oba 1352–1358

Xanthine oxidoreductase

Differential regulation of hypoxanthine and xanthine by obesity in a general population, Furuhashi 878–887

Zinc transporter 8 autoantibodies

Zinc transporter 8 autoantibodies complement glutamic acid decarboxylase and insulinoma‐associated antigen‐2 autoantibodies in the identification and characterization of Japanese type 1 diabetes, Kawasaki 1181–1187

